# Mismatch Negativity as an Indicator of Cognitive Sub-Domain Dysfunction in Amyotrophic Lateral Sclerosis

**DOI:** 10.3389/fneur.2017.00395

**Published:** 2017-08-15

**Authors:** Parameswaran Mahadeva Iyer, Kieran Mohr, Michael Broderick, Brighid Gavin, Tom Burke, Peter Bede, Marta Pinto-Grau, Niall P. Pender, Russell McLaughlin, Alice Vajda, Mark Heverin, Edmund C. Lalor, Orla Hardiman, Bahman Nasseroleslami

**Affiliations:** ^1^Academic Unit of Neurology, Trinity Biomedical Sciences Institute, Trinity College Dublin, Dublin, Ireland; ^2^Department of Neurology, Beaumont Hospital, Dublin, Ireland; ^3^Department of Psychology, Beaumont Hospital, Dublin, Ireland; ^4^Trinity College Institute of Neuroscience, Trinity College Dublin, The University of Dublin, Dublin, Ireland; ^5^Trinity Centre for Bioengineering, Trinity College Dublin, The University of Dublin, Dublin, Ireland; ^6^Department of Biomedical Engineering and Department of Neuroscience, University of Rochester, Rochester, NY, United States

**Keywords:** amyotrophic lateral sclerosis, auditory mismatch negativity, neuronal networks, EEG, cognition, phenotyping

## Abstract

**Objective:**

To evaluate the utility of mismatch negativity (MMN), a neurophysiologic marker of non-motor cognitive processing, in amyotrophic lateral sclerosis (ALS).

**Methods:**

89 patients, stratified into 4 different phenotypic presentations of ALS (67 spinal-onset, 15 bulbar-onset, 7 ALS-FTD, 7 *C9ORF72* gene careers), and 19 matched controls underwent 128-channel EEG data recording. Subjects were presented with standard auditory tones interleaved with pitch-deviant tones in three recording blocks. The MMN response was quantified by peak amplitude, peak delay, average amplitude, and average delay, 100–300 ms after stimuli. 64 patients underwent cognitive screening using the Edinburgh Cognitive and Behavioural ALS Screen (ECAS), and 38 participants underwent contemporaneous cognitive assessment using the Stroop Color–Word Interference test (CWIT), which measures attention shift, inhibitory control, and error monitoring.

**Results:**

The MMN response was observed in frontal and frontocentral regions of patient and control groups. Compared to controls, waveforms were attenuated in early onset, and the average delay was significantly increased in all of the ALS subgroups, with no significant difference between subgroups. Comparing with the control response, the ALS MMN response clustered into four new subgroups characterized by differences in response latency. The increased average delay correlated with changes in the Stroop CWIT; however, it did not show a direct relationship with age, gender, traditional phenotypes, revised ALS Functional Rating Scale, or ECAS scores.

**Conclusion and significance:**

The MMN response in ALS patients reflects the cognitive dysfunction in specific sub-domains, as the new patient subgroups, identified by cluster analysis, do not segregate with existing clinical or cognitive classifications. Event-related potentials can provide additional quantitative neurophysiologic measures of impairment in specific cognitive sub-domains from which it may be possible to generate novel biologically relevant subgroups of ALS.

## Introduction

Amyotrophic lateral sclerosis (ALS) is an age-related neurodegenerative disorder (1) characterized by a combination of upper and lower motor neuron impairment (2, 3). Preclinical assessment of potential new therapeutics has been based primarily on the concept of selective vulnerability of individual motor neurons, and outcome metrics in human clinical trials have focused on survival, functional activity, and muscle strength (4). However, recent evidence demonstrates the presence of extensive extra-motor involvement in some patients with ALS (1, 5), and the discovery in 2011 of a hexanucleotide expansion in *C9ORF72* as a causative factor in both ALS and FTD (6) indicate that these two conditions are biologically linked. The presence of patterns of progressive cognitive and behavioral changes suggest that different frontotemporal and frontostriatal neuroanatomical pathways can be affected in ALS, manifesting as executive impairment, and patterned behavioral change including apathy, rigidity, or disinhibition, respectively (7, 8). Taken together with progressive motor decline, the clinical manifestations of ALS can be construed as a progressive disintegration of neural networking in motor and non-motor network domains (9). Observed changes in connectivity in ALS by fMRI, and magneto-/electro-encephalography (e.g., resting-state connectivity assessed using spectral cortico-cortical coherence analysis) support this construct (10, 11). Further elaboration of the nature, extent, and clinical correlates of network disruption is likely to provide useful data from which non-invasive biomarkers of disease pathophysiology could be discovered.

Task-related neural activation such as event-related/evoked potentials (12, 13) provide information about brain networks underlying somatosensory responses, visual responses, and complex responses that measure error monitoring and attention during specific functional tasks. In this context, the auditory mismatch negativity (MMN) response (14), which is a measure of the difference between standard and unexpected deviant auditory event-related potentials (ERPs), is a well-described physiological response to an involuntary attention shift (e.g., auditory oddball paradigm), generated by a multi-node network (15), and visible in EEG recorded over frontal scalp regions (16). Though the traditional standard and deviant stimuli vary in their tones, MMN can be elicited by subtle changes in phonemes, semantics, grammar, and other linguistic characteristics (17).

The different components in the MMN ERP can be broadly divided into early and late responses. Early responses are those occurring within 100 ms after stimulus, and late responses are those occurring thereafter (16, 17). Early responses are related to the physical character of the stimulus, while late ones are endogenous and reflect cognitive aspects of auditory processing (16, 17). In general, reduction in the amplitude of the MMN response parallels increased connectivity and reduced inhibitory control, especially in the frontal generators (18). Regardless of the underlying mechanisms, MMN is a recognized measure of attentional systems in a broad range of neuropsychiatric conditions such as schizophrenia (19) and in subclinical psychotic symptoms (20). Taken together, these findings suggest that MMN could be also a suitable biomarker of extra-motor manifestations of ALS, with particular reference to impairment of networks involved in executive functions such as involuntary attention shift and response selection (21).

Previous studies have demonstrated that the MMN amplitude and duration correlates with changes in various neurologic conditions, including FTD (22) and subclinical psychosis (23). The MMN response in ALS has been reported to show both normal waveforms, and suppressed amplitudes or increased latencies (13, 24–27).

To further elucidate the MMN response in ALS and to determine whether clinically defined ALS subgroups differ in this response, we used a large sample size of well-characterized ALS patients divided into five clinical subgroups, and a cohort of age- and gender-matched controls. We measured the MMN response in ALS patients compared to controls and sought to determine whether differences in this response correlated with attention-based executive functions, with the purpose of assessing the utility of MMN as a biomarker of decline in cognitive sub-domains that could be harnessed to identify sub-phenotypes of ALS.

## Materials and Methods

### Ethical Approval

Approval for this study was obtained from the ethics committee of Beaumont Hospital, Dublin, Ireland [Research Ethics Committee (REC) reference: 13/102] and the Tallaght Hospital/St. James’s Hospital Joint REC (REC reference: 2014 Chairman’s Action 7, CRFSJ 0046) for St. James’s Hospital, Dublin, Ireland. The experimental procedure conformed to the Declaration of Helsinki. All participants, including the patients and healthy controls, provided written informed consent before taking part in the experiments.

### Participants

#### Patient Recruitment

Patient recruitment was undertaken from ALS patients attending the National ALS specialty clinic in Beaumont Hospital. Healthy controls were recruited from neurologically normal, age-, and sex-matched individuals recruited as part of an existing cohort study of cognition in ALS.

#### Inclusion Criteria

Patients were recruited into four subgroups as follows: bulbar-onset ALS, spinal-onset ALS, ALS-FTD, and those carrying the *C9ORF72* repeat expansion. All ALS patients were within the first 18 months since diagnosis and fulfilled the El Escorial diagnostic criteria for possible, probable, or definite ALS.

#### Exclusion Criteria

Patients diagnosed with primary lateral sclerosis, progressive muscular atrophy, flail arm/leg, transient ischemic attack, multiple sclerosis, stroke, epilepsy, seizure disorder, brain tumors, structural brain diseases, other degenerative brain diseases, and other comorbidities (e.g., human immunodeficiency virus) were excluded.

#### Demographics of Patients and Controls

A total of 89 patients (f/m: 26/63; age: 60.6 ± 11.6 years in the range 32–82), 67 patients with spinal-onset ALS, 15 patients with bulbar-onset ALS, and 7 with ALS/FTD, along with 19 healthy controls (f/m: 9/10; age: 61.0 ± 22.5 in the range 30–75) were recruited.

Seven patients carried a hexanucleotide expansion in *C9ORF72* (Table 1). The ALS Functional Rating Scale (ALSFRS-R) values collected from 87 of the patients within 1 month of EEG data acquisition ranged from 13 to 48, with a mean (±SD) of 36.5 ± 7.7 (Table 1). 17 patients had a known family history of at least one first or second degree relative with ALS (28). 78 patients were taking riluzole with 76.0 ± 60.0 days (median ± IQR) past since starting the medication. 64 patients (see Table 2) underwent cognitive screen using the Edinburgh Cognitive and Behavioural ALS Screen (ECAS) (29), and a sub-cohort (38 patients) underwent more detailed neuropsychological assessment within 2 weeks of EEG, including the *Stroop Color-Word interference test (CWIT)* (Table S1 in Supplementary Material).

**Table 1:** Age, gender, and diagnosis status of the participants.

Group		*n*	Male	Female	Age (years)^a^	Time since diagnosis (days)^a^	ALSFRS-R
Control		19	10	9	61.0 ± 22.5	–	–
ALS	All	89	63	26	60.6 ± 11.6	275 ± 373	36.5 ± 7.7^(^*^n^ *^= 86)^
	Spinal	67	50	17	60.0 ± 11.9	286 ± 301	36.5 ± 7.0^(^*^n^ *^= 64)^
	Bulbar	15	9	6	58.2 ± 9.4	216 ± 267	35.2 ± 10.7^(^*^n^ *^= 15)^
	ALS-FTD	7	4	3	71.5 ± 6.9	289 ± 301	38.9 ± 7.1^(^*^n^ *^= 7)^
	*C9ORF72*+	7	3	4	58.1 ± 9.4	336 ± 167	39.7 ± 8.6^(^*^n^ *^= 9)^
	*C9ORF72*−	63	48	15	61.9 ± 11.5	288 ± 412	36.2 ± 7.6^(^*^n^ *^= 64)^

*^a^Mean ± SD*.

*ALS, amyotrophic lateral sclerosis; ALSFRS-R, Revised ALS Functional Rating Scale*.

**Table 2:** ECAS measures, age, gender, and diagnosis status of the patient subgroups with available ECAS information.

Group		*n*	Male	Female	Age (years)^a^	Time since diagnosis (days)^a^	ECAS (language)	ECAS (fluency)	ECAS (executive)	ECAS (memory)	ECAS (visuospatial)
ALS	All	64	47	17	58.5 ± 11.7	283 ± 423	26.0 ± 2.4 (*n* = 64, 78% normal)	18.0 ± 3.0 (*n* = 60, 83% normal)	35.4 ± 7.6 (*n* = 61, 83% normal)	15.9 ± 4.6 (*n* = 63, 82% normal)	11.8 ± 0.7 (*n* = 64, 93% normal)
	Spinal	51	39	12	58.8 ± 12.3	304 ± 452	25.9 ± 2.6 (*n* = 51, 78% normal)	17.9 ± 3.2 (*n* = 47, 82% normal)	35.5 ± 7.7 (*n* = 50, 86% normal)	15.6 ± 4.9 (*n* = 51, 80% normal)	11.7 ± 0.8 (*n* = 51, 92% normal)
	Bulbar	13	8	5	57.0 ± 9.5	200 ± 280	26.2 ± 1.2 (*n* = 13, 76% normal)	18.3 ± 2.4 (*n* = 13, 84% normal)	35.1 ± 7.7 (*n* = 11, 72% normal)	17.0 ± 2.7 (*n* = 12, 91% normal)	12.0 ± 0.0 (*n* = 13, 100% normal)
	Cognitive/ALS-FTD	0									
	C9Orf72+	4	2	2	59.6 ± 10.4	488 ± 519	23.8 ± 5.4 (*n* = 4, 75% normal)	14.5 ± 6.0 (*n* = 4, 75% normal)	27.7 ± 12.7 (*n* = 3, 66% normal)	11.3 ± 7.8 (*n* = 4, 50% normal)	11.0 ± 2.0 (*n* = 4, 75% normal)
	C9Orf72−	51	37	14	59.0 ± 11.8	297 ± 447	26.4 ± 1.6 (*n* = 51, 76% normal)	18.2 ± 2.8 (*n* = 48, 81% normal)	35.9 ± 6.6 (*n* = 49, 85% normal)	16.0 ± 4.4 (*n* = 50, 82% normal)	11.9 ± 0.4 (*n* = 51, 96% normal)

*^a^Mean ± SD*.

*ALS, amyotrophic lateral sclerosis; ECAS, Edinburgh Cognitive and Behavioural ALS Screen*.

### Experimental Paradigm

The experiment was divided into three 8-min recording blocks, allowing for rest between blocks. Subjects were seated and asked to attend to a silent, black and white film for the duration of the experiment while auditory tones were played through headphones and their neural responses recorded.

#### EEG Acquisition

128-channel EEG data were filtered over the range of 0–134 Hz and digitized at 512 Hz using the BioSemi^®^ Active Two system (BioSemi B.V., Amsterdam, Netherlands). Recordings were conducted in dedicated laboratories in the University of Dublin and St. James’s Hospital, Dublin.

#### Auditory Stimuli

The standard and deviant auditory stimuli were generated by Presentation^®^ software (NeuroBehavioral Systems, Inc., Berkeley, CA, USA) on a PC and delivered to the subjects through HD650 headphones (Sennheiser, Wedemark, Germany). The frequencies of standard and deviant tones were 720 and 800 Hz, respectively, giving deviant tones a slightly higher pitch (i.e., a frequency mismatch paradigm). The duration of both standard and deviant tones was 150 ms, and their interstimulus interval was 833 ms. Deviant tones constituted about 10% of the presented stimuli.

### Cognitive Performance

The ECAS scores were used to screen for overall cognitive change associated with ALS (29). Age- and education-matched normative data (8) for each participant were used to establish cognitive status (29).

The CWIT (30, 31) is a well-established and common neuropsychological test, which measures multiple dimensions of executive control including error monitoring, working memory, selective attention, and inhibitory control (32). This test is composed by two tasks: (1) *Part-A* (the “priming” task), where patients are presented with a list of color word names that are printed in the intuitive color, i.e., red was printed in red ink, and asked to read the printed words as quickly as possible, while being timed; (2) *Part-B* (the inhibitory/interference task), where patients are presented with another list of color words in which the ink color and word are incongruent, i.e., red is printed in blue, and the patient is asked to name as quickly as possible the *color of ink* in which each word is printed. In doing so, the action of reading the printed word is inhibited. An algorithm is then applied to the data to reduce the potential confounding effects of motor involvement, known as the Stroop executive factor (5). All participants who completed this test had normal color vision. Three participants were not administered the Stroop CWIT, due to self-reported color-blindness.

### Data Analysis

#### EEG Signal Processing

Signal analysis was performed in MATLAB^®^ (The Mathworks, Inc., Natick, MA, USA), using custom written scripts for EEGLAB (33) and ADJUST (34). Data were high- and low-pass filtered at cutoff frequencies of 0.3 Hz (dual-pass 5th order Butterworth filter) and 35 Hz (dual-pass 117th order equiripple finite impulse response filter), respectively. Subsequently, episodes of heavily contaminated EEG recordings were removed by visual inspection, and the data were epoched to include 100 ms before and 500 ms after the onset of the auditory stimuli. Artifacts were removed using independent component analysis (ICA) *via* decision rules given by ADJUST. In subjects with small auditory-evoked potentials (AEPs), the automatically identified (as artifact or signal) ICA components were reinspected visually to remove the remaining artifactual components and to include incorrectly rejected components. The presence of AEPs was the criterion for inclusion of the analyzed dataset for MMN analysis. The common-average referenced EEG epochs were then averaged separately for standard and deviant trials to yield the standard and deviant AEPs, as well as their difference (MMN). A dual-pass 5th order Butterworth low-pass filter (cutoff frequency: 15 Hz) was used to eliminate the remainder of potential artifactual components in the analysis and for better visualization of the results. Using a baseline of 100 ms (found to maximize the signal to noise ratio: AEP power before the event divided by the considered baseline’s signal power), the AEPs were baseline-adjusted for analysis.

#### Statistics

The statistical significance of the MMN waveform (difference from 0) at different electrodes and each time-point after the event was tested in the control and patient groups using Wilcoxon’s Signed Rank test (α = 0.05). The significance of between-group differences of the MMN waveforms was similarly tested using the Mann–Whitney *U* test (α = 0.05). The multiple comparison correction factor was taken as the number of dominant principal components (as previously used for ERPs) (35) that accounted for the MMN response across electrodes and time-points between 100 and 300 ms. Further analysis of changes in multidimensional EEG across time-points and channels was performed using empirical Bayesian inference (EBI) (36).

#### Measures of Change in MMN Response

After calculating the time interval in which MMN was significantly different between different patient groups and controls, the MMN amplitudes recorded at these time-points were averaged for each participant and a single MMN amplitude value was obtained and then compared across ALS subgroups (Kruskal–Wallis test, α = 0.05) to test for the effect of subgroups (but not as a retest between controls and patients). Additionally, four waveform features of the MMN response, namely the amplitude of the peak, delay of the peak, integrated area (as an average amplitude), and center of area (which acts as the “average delay”) were assessed between 100 and 300 ms. Adaptive false discovery rate (FDR) (37, 38) was used to correct for the five comparisons (the average MMN and four waveform features) at *q* = 0.05.

#### Cognitive Correlation

The MMN features that showed significant between-group differences were tested for correlation against the Stroop CWIT.

#### Patient Clusters

The clustering of patients based on the MMN measures was performed using *k*-mean clustering algorithm (39), where the optimal number of clusters was found using the optimal Davies–Bouldin measure of clusters separation (40).

## Results

### Presence of MMN

**Figure 1:**
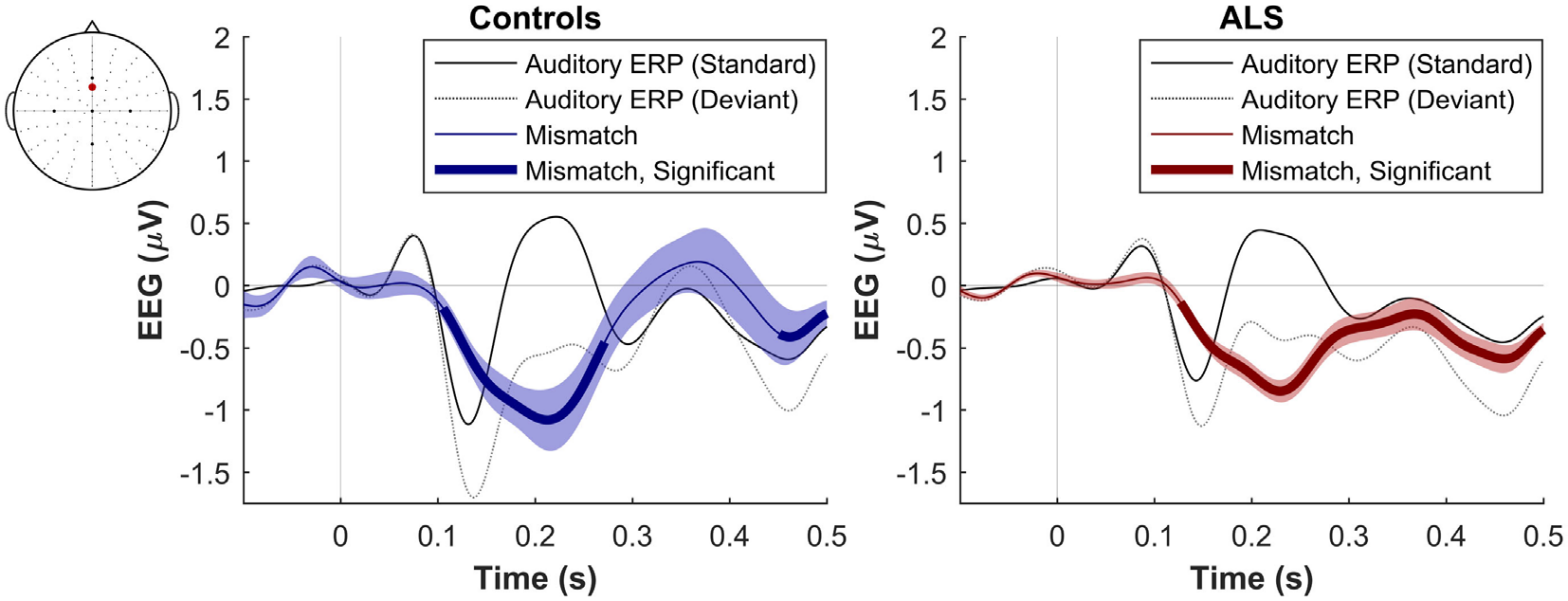
Presence of robust mismatch negativity responses in both patient and control groups. The auditory event-related potentials (ERPs), as well as their difference are shown. The shades indicate the SEM. See text for statistics and significance (*p* < 0.05).

**Figure 2:**
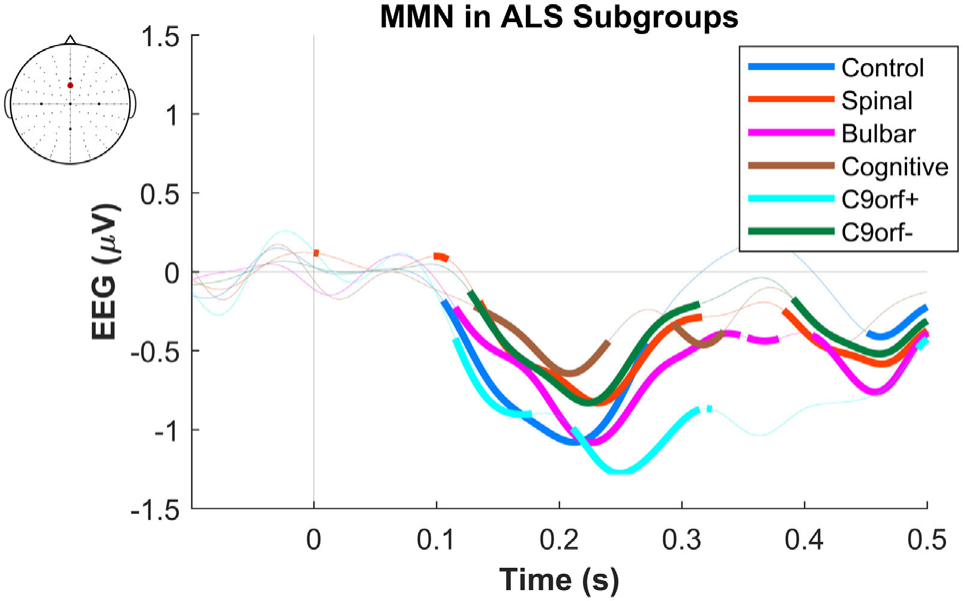
Presence of robust mismatch negativity (MMN) responses in individual patient subgroups of amyotrophic lateral sclerosis (ALS). The MMN waveforms in controls (*n* = 19), spinal-onset ALS (*n* = 67), bulbar-onset ALS (*n* = 15), ALS-FTD (*n* = 7), ALS patients with *C9ORF72* repeat expansion, and with no *C9ORF72* repeat expansion are shown. The thick curves indicate the significant responses (*p* < 0.05).

The maximum MMN in midline electrodes for the control group was taken as a reference for the presence of the MMN. The maximum and average negative amplitude of between 100 and 300 ms was recorded at an electrode between Fz and FCz. The MMN became significant in the control group from 105 ms and lasted up to 271 ms (Wilcoxon’s Signed Rank test, *p* < 0.05, *n* = 19) (Figure 1). The difference from controls in MMN response was statistically significant in each of the individual ALS subgroups: spinal-onset, bulbar-onset, ALS-FTD, C9ORF72+, and C9ORF72− (Figure 2). For the ALS groups, the MMN became significant from 127 ms and persisted up to 500 ms (Wilcoxon’s Signed Rank test, *p* < 0.05) (Figure 1). As 82 and 93% of the variance for multichannel average MMN between 100 and 300 ms was accounted for by only one principal component, the multiple comparison correction factor for significance testing of MMN waveforms was considered as 1. The significant time segments of the MMN were then analyzed in controls and ALS patients using EBI: in controls, the MMN was significant between 102 and 268 ms, and in ALS patients it was significant between 125 and 500 ms across all patients, thus confirming the statistical inference.

### Differences between Controls and Patients

**Figure 3:**
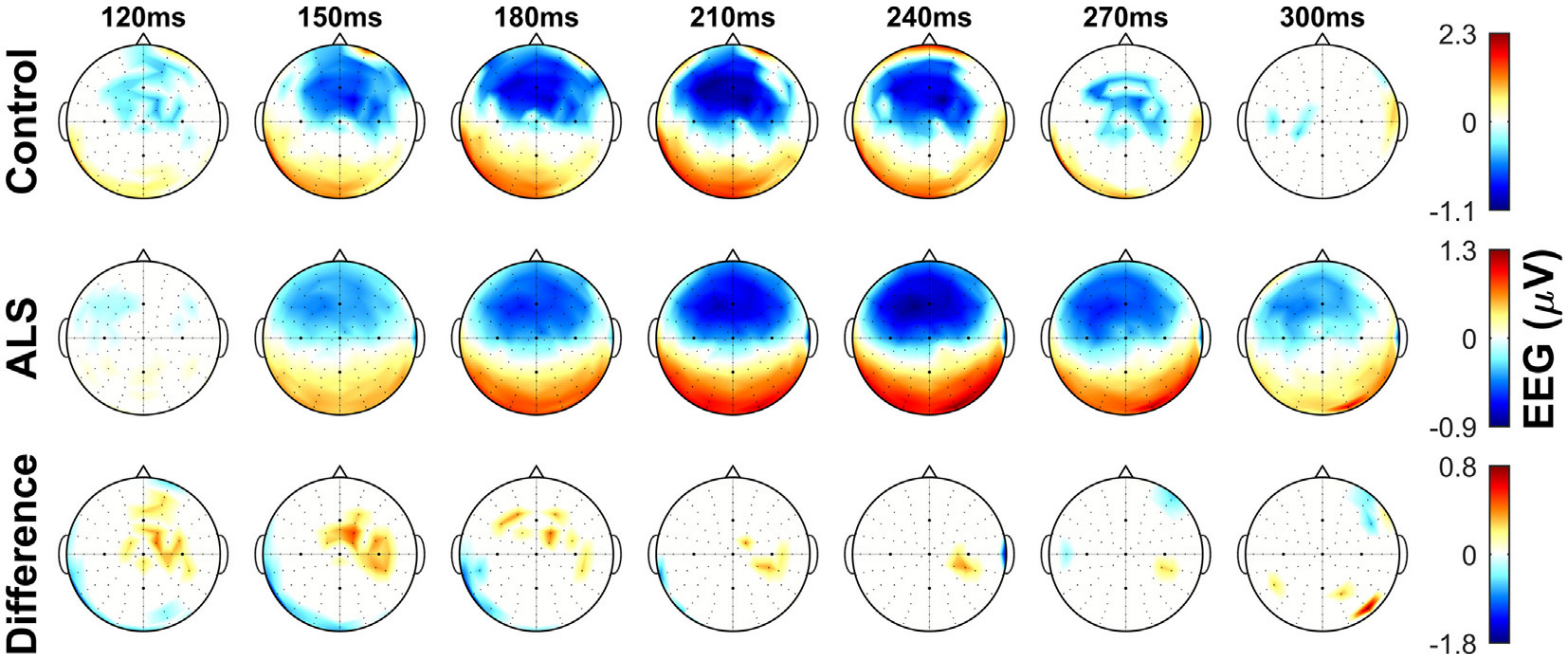
Topographic maps showing the mismatch negativity (MMN) in controls and amyotrophic lateral sclerosis (ALS) patients, as well as the between-group differences in frontocentral regions. Notice the overall time shift of MMN in the ALS group. Non-significant (*p* > 0.05) values of MMN in each group and their between-group difference are shown as 0. See text for statistics and significance.

**Figure 4:**
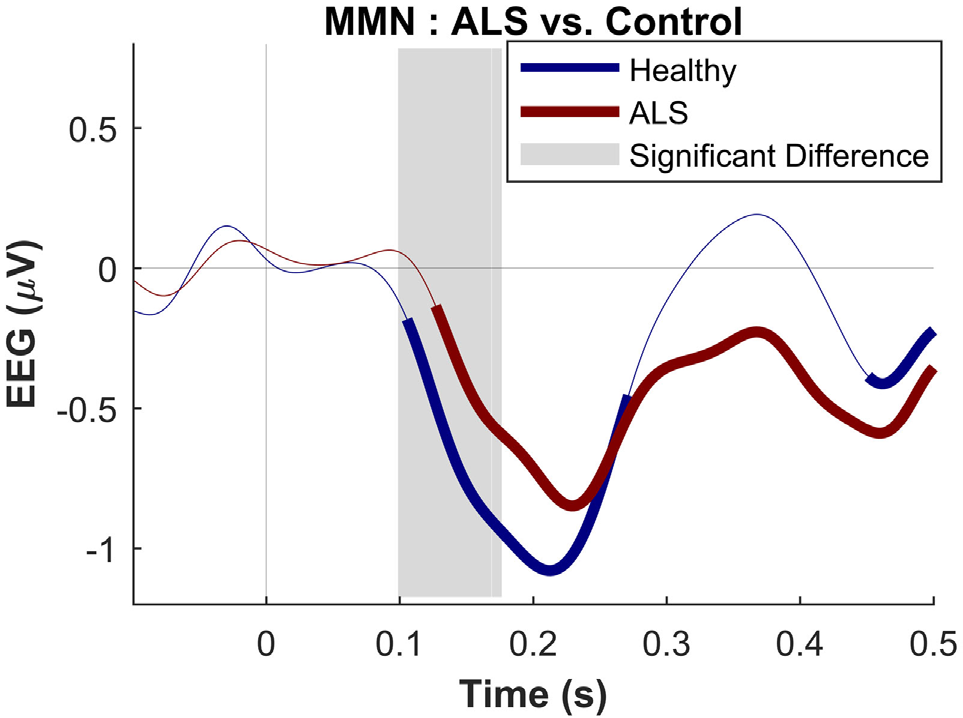
Presence of robust mismatch negativity responses in both patient and control groups. The auditory event-related potentials (ERPs) as well as their difference are shown. The shades indicate the SEM. See text for statistics and significance (*p* < 0.05)

**Figure 5:**
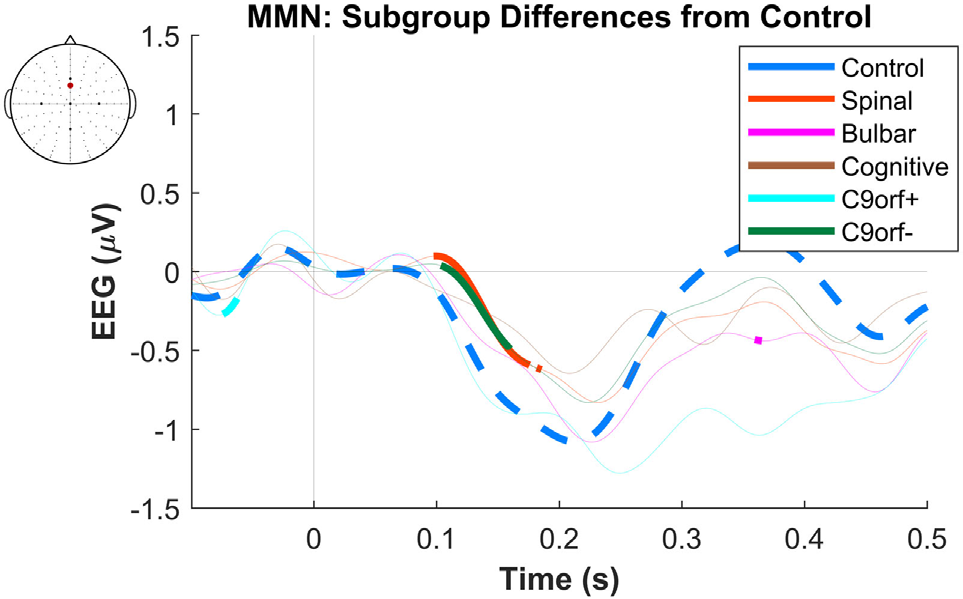
Difference of early average mismatch negativity (MMN) between controls and individual amyotrophic lateral sclerosis subgroups. The between-group differences correspond to each time-point of EEG in a frontocentral electrode located between FCz and Fz. The thick curves indicate time windows where the differences against the control waveforms are significant. See text for statistics and significance (*p* < 0.05).

The dominant presence of the MMN for both patients and controls was in the frontal and frontocentral electrodes (Figure 3). The time windows where the MMN shows a statistically significant decrease in the combined ALS patient cohort compared to controls was 99–173 ms (Mann–Whitney *U* test, *p* < 0.05, *n*_1_ = 19, *n*_2_ = 89) (Figure 4), with the most prominent difference between patients and controls in the spinal and *C9ORF72*-negative sub-cohorts (Figure 5).

We then analyzed the abstract features of the MMN waveform in the ALS cohort and compared with the control cohort.

**Figure 6:**
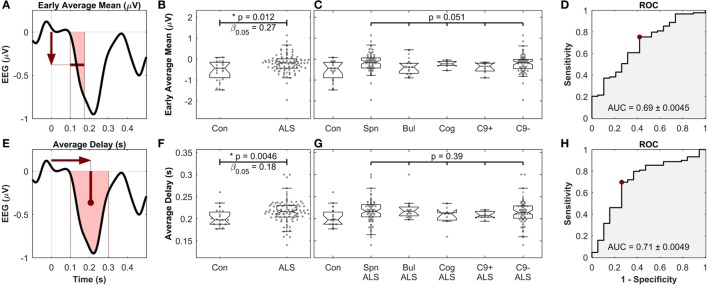
Early average mean [top, **(A–D)**] as well as the average delay [bottom, **(E–H)**] of mismatch negativity (MMN), showing significant differences between controls and amyotrophic lateral sclerosis (ALS) patients, but not between ALS subgroups. **(A,E)** Schematic of the definition of the MMN waveform measures. **(B,F)** Comparison between healthy controls and all ALS patients. **(C,G)** Comparison between ALS subgroups. “Con,” “Spn,” “Bul,” “Cog,” “C9+,” and “C9−” stand for control, spinal, bulbar, cognitive/ALF-FTD, *C9ORF72* positive and *C9ORF72* negative subgroups, respectively. **(D,H)** ROC curve, comparing the discriminatory power, the optimal level of sensitivity–specificity (red dot), as well as the area under the curve (AUC), achieved for linear discrimination of controls and ALS patients of each measure. See text for methods and statistics.

To obtain a single average value for each patient, the MMN amplitudes were averaged in the time segment 99–173 ms (where the waveforms were significantly different between controls and patient subgroups) to find the “early average MMN.” The early average MMN was not significantly different across the five ALS subgroups (Figures 6A–D).

The other four MMN waveform measures (peak amplitude, peak delay, average amplitude, and average delay) were then investigated after the early average MMN. The average MMN delay was significantly longer in ALS patients (217 ms ± 26.4), compared to controls (199 ms ± 29.6) (Mann–Whitney *U* test, *p* = 0.0046, *n*_1_ = 19, *n*_2_ = 89, α_FDR_ > 0.01, power = 1 − β_0.05_ = 0.82). Comparison of the average MMN delay between individual ALS subgroups and controls yielded significantly longer delay in spinal, bulbar, and C9ORF72− groups (*p* < 0.05), but no differences were detected between these subgroups (*p* = 0.39, Kruskal–Wallis test, *n_i_* = 68, 15, 7, 7, 63) (Figures 6E–H). The other measure, namely the peak delay, peal amplitude, and average amplitude, did not show any significant changes.

### Effects of Age, Gender, and Neuropsychological Status

The average MMN delay was not different between male and female patients or controls, and there was no correlation (Spearman’s rank correlation) between the average delay and patients’ age (*p* = 0.08) or time since diagnosis (*p* = 0.23). Neither was the MMN delay dependent on the use of riluzole: 216 ± 25 ms for those using riluzole versus 228 ± 36 ms for those not using the drug (Mann–Whitney *U* test, *p* = 0.08, *n*_1_ = 77, *n*_2_ = 10). Correlation between the time patients were on riluzole and the average MMN delay was not significant (Spearman’s Rank Correlation, rho = 0.07, *p* = 0.54, *n* = 77). A significant correlation at group level was noted in the ALS cohort between the average MMN delay and the priming trial time of the Stroop CWIT (*r* = 0.43, *p* = 0.0073, *n* = 38, Spearman’s rank correlation coefficient, α_FDR_ > 0.025, power = 1 − β_0.05_ = 0.87), but not between the average MMN delay and the time taken to complete the inhibition trial of the Stroop CWIT.

### Clustering Based on MMN Delay

**Figure 7:**
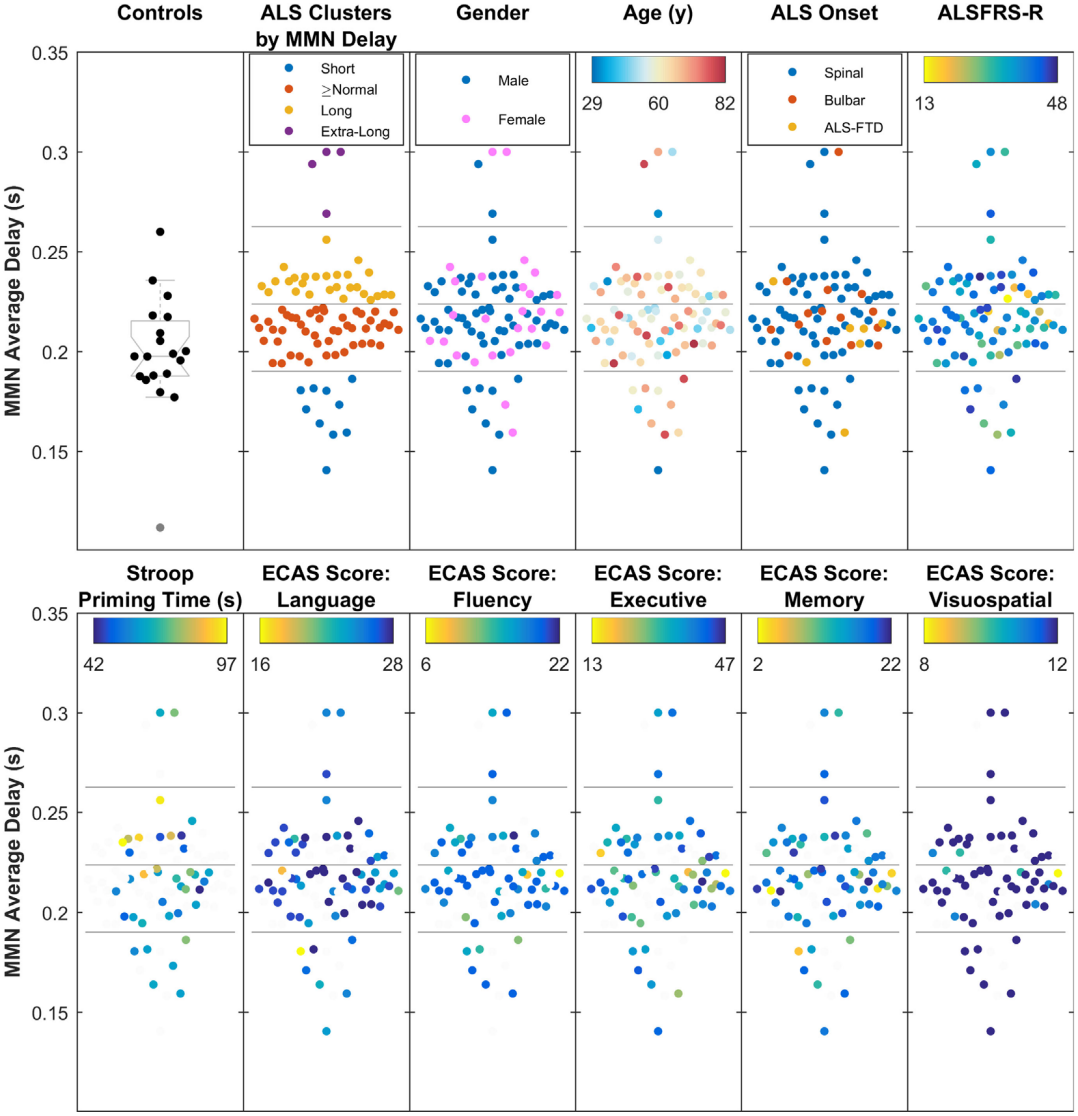
The four clusters of amyotrophic lateral sclerosis (ALS) patients, based on the average mismatch negativity (MMN) delay (short, above normal, long, and extra-long delays), showing moderate relationship with Stroop Color–Word Interference task (i.e., cognition), but no overlap with traditionally defined phenotypes, age, gender, ALS Functional Rating Scale (ALSFRS-R), or five main Edinburgh Cognitive and Behavioural ALS Screen (ECAS) scores. The four subgroups, found through *k*-mean clustering of the average MMN delay are shown against healthy controls.

Cluster analysis of the data from MMN average delay using data from all ALS patients revealed four subgroups of patients (where the MMN delay was short, equal-to/above normal, long, and extra-long). These neurophysiologic clusters (Figure 7) did not correlate with the age, gender, traditional phenotypes, or ALSFRS-R.

Stroop priming times (Table S1 in Supplementary Material), which showed correlation with the average delay, also correlated with subcluster analyses (Figure 7).

Cognitive status based on ECAS scores (see Table 2) did not show significant correlations with the average delay of MMN. However, it must be noted that the majority of ALS patients (85%) from whom ECAS data were available scored within the normal range for each sub-domain assessed (refer to Table 2 for further details).

## Discussion

Our findings confirmed the occurrence of MMN response in a frontal and frontocentral topographic distribution as previously described (18) with ranges for the MMN response consistent with that of previously published studies (17, 41), thus validating our experimental design and data analyses.

We found that the N2a component of the ERP responses discriminated between ALS and controls. This component is elicited by any change in repetitive background stimulation and is likely to represent an involuntary process that identifies change in a stimulus pattern (42, 43). Although analysis of this component has traditionally been based on the characteristics of the waveform peak rather than on average delay, we focused on the latency, and the amplitude of the early negativity response between 99 and 173 ms, which in our study were different between ALS and controls. We did not detect any significant change in other waveform features (peak amplitude, peak delay, average amplitude).

As the purpose of our study was to determine whether the MMN response correlated with clinical sub-phenotype in ALS, we selected four clinically relevant subgroups characterized by site of onset, presence of FTD, and presence of the *C9ORF72* repeat expansion. While we noted that the MMN response differed between controls and ALS patients, we did not detect any discriminatory aspects within the MMN that correlated with any of the specific clinical disease sub-phenotypes.

Mismatch negativity is an already established ERP component measure that has been proved useful in assessing abnormal neural responses in a range of clinical conditions (18, 19, 23). In our study, we noted that MMN features correlated with changes in specific cognitive sub-domains (i.e., attentional shift among patients with ALS, quantified by Stroop CWIT), but not with other cognitive sub-domains assessed by the ECAS, and which are relevant to ALS (e.g., language, fluency, executive).

This finding could be explained by the nature and utility of the ECAS, which is a screening tool with good sensitivity and specificity at detecting cognitively abnormal ALS patients when overall score is considered, and when age- and education-matched cutoff scores are applied (8). The ECAS is designed to screen for changes in cognitive domains that have been demonstrated to be affected in ALS (ALS-specific: language, executive function, and verbal fluency), and cognitive domains that are not specific for ALS (memory and visuospatial abilities), through a series of subtests. Although interpretation of ALS-specific domain scores is adequate, performance on individual subtests or non-specific domains on the ECAS cannot be reliably interpreted individually due to their low sensitivity (8). Accordingly, the absence of a significant correlation between MMN and ECAS is most likely a function of the nature of the ECAS as a screening instrument rather than an extensive neuropsychological battery (5, 8, 44).

In this study, the Stroop CWIT data significantly correlated with the MMN. The Trenerry et al. version of the Stroop test was specifically chosen *a priori*, as it has been repeatedly shown to be a valid, reliable, and psychometrically robust measure of executive dysfunction in ALS, which can control for motor disability in ALS. Stroop test could be seen as a test of efficacy of attentional systems while performing a task that requires executive attention.

The neurophysiologic measure of cognitive network impairment that we have identified by cluster analysis did not correlate with the established clinical subgroups of ALS. This is not entirely surprising. Source analysis of the MMN (45) suggests that MMN is initially processed in the temporal lobe and that the later cognitive component is generated by an involuntary attention-switching mechanism that involves the inferior and superior frontal gyrus, anterior insula, and anterior cingulate cortex (which are key nodes of salience network) (46, 47). Using resting-state EEG, we have previously shown evidence of increased connectivity in the anterior insular cortex and anterior cingulate cortex among ALS patients compared to controls (11), and our finding of difference in the MMN response latency between ALS and controls is congruent with this. This is indirectly suggestive of an altered connectivity in components of the salience network of the brain, which overlaps with and includes the key generators of the MMN response. Although this interpretation requires validation by connectivity analysis in the source space (20, 48), it is consistent with our observation of changes in MMN features and its correlation with cognitive performance in the Stroop CWIT test. The study further shows that MMN could potentially act as a neurophysiologically captured component of attentional network impairment in ALS. This is neither an arbitrary secondary effect of neurodegeneration nor a pharmacological effect of riluzole (49).

Failure of ALS therapeutic trials is frequently associated with incomplete patient stratification, most of which to date has been based on traditional phenotypic classification. There is now a recognized need to develop enhanced biomarkers that can further subcategorize and monitor patients within more homogeneous subgroups. We undertook *post hoc* cluster analysis of the MMN response and have identified four distinct subgroups within the ALS cohort, suggesting the presence of neurophysiologic heterogeneity. These latent ALS subgroups do not have an obvious clinical correlate, at least based on clinical phenotype and cognitive screening using ECAS. However, it is possible that these subgroups reflect the neurophysiologic correlates of more subtle cognitive and behavioral sub-phenotypes, specifically in the sub-domain of involuntary attentional shift, not captured by ECAS, but discernible using more extensive cognitive and behavioral batteries (50). While this subclassification based on MMN response is an early preliminary step, it can be seen as a starting point for novel stratification strategies that do not follow the traditional classification of ALS spectrum based on the anatomic origin.

Our study is limited by the cross-sectional design, and the inclusion of patient sub-cohorts of different sizes, although our statistical methodology accounted for this variability. Although our observation of an increased delay of MMN in ALS is adequately powered, ROC analysis suggests that this measure does not currently have strong discriminatory potential for use as a diagnostic disease biomarker in ALS when compared with controls. Notwithstanding the limitations, our findings add further neurophysiologic evidence of probable early and heterogeneous network disruption in ALS and suggest that additional source analysis of the MMN, coupled with detailed further cognitive and behavioral characterization could uncover sub-phenotypes of ALS characterized by differential network disruption (50). Moreover, longitudinal evaluation of the MMN waveform at individual level could be exploited as a non-invasive quantitative biomarker of disruption of non-motor networks.

In conclusion, the MMN response in ALS patients reflects the cognitive dysfunction in specific sub-domains, as the new patient subgroups (identified by cluster analysis) do not segregate with existing clinical or cognitive classifications. ERPs can provide additional quantitative neurophysiologic measures of impairment in specific cognitive sub-domains from which it may be possible to generate novel biologically relevant subgroups of ALS that could be utilized in the evaluation of novel therapeutics.

## Ethics Statement

Approval for this study was obtained from the ethics committee of Beaumont Hospital, Dublin, Ireland (REC reference: 13/102) and the Tallaght Hospital/St. James’s Hospital Joint Research Ethics Committee (REC) (REC reference: 2014 Chairman’s Action 7, CRFSJ 0046) for St. James’s Hospital, Dublin, Ireland. The experimental procedure conformed to the Declaration of Helsinki. All participants, including the patients and healthy controls, provided written informed consent before taking part in the experiments.

## Author Contributions

PI: hypothesis development, data collection, drafting manuscript, patient recruitment, and assistance in data analysis. KM and MB: data collection, data analysis, and manuscript editing. BG and PB: data collection and manuscript editing. TB: data collection, neuropsychological assessment, and manuscript drafting/editing. MP-G and NP: neuropsychological assessment and manuscript drafting/editing. RM: genetic data analysis. AV: genetic data analysis and patient management. MH: manuscript drafting/editing and data collection. EL: hypothesis development and data analysis. BN: data analysis, statistical analysis, editing and revising manuscript, and data collection. OH: hypothesis development, data collection, manuscript drafting and revision, as well as project overview.

## Conflict of Interest Statement

PI, KM, MB, BG, MP-G, NP, RM, AV, MH, EL, BN, and TB: no disclosures. OH has received speaking honoraria from Janssen Cilag, Biogen Idec, Sanofi Aventis, Novartis, and Merck-Serono. She has been a member of advisory panels for Biogen Idec, Allergen, Ono Pharmaceuticals, Novartis, Cytokinetics, and Sanofi Aventis. She serves as Editor-in-Chief of *Amyotrophic Lateral Sclerosis and Frontotemporal Dementia*.
