# Supplementary material for: Mismatch Negativity as an Indicator of Cognitive Sub-Domain Dysfunction in Amyotrophic Lateral Sclerosis
Source: Front Neurol. 2017 Aug 15;8:395. doi: 10.3389/fneur.2017.00395 (PMC5559463; doi:10.3389/fneur.2017.00395)
Supplement: Supplementary file 1 [file Table_1.DOCX]

**Table S1.** Stroop Priming Times, age, gender and diagnosis status of the patient subgroups with available Stroop data.

| **Group** |  | **n** | **Male** | **Female** | **Age**  **(y)*** | **Time since Diagnosis**  **(days)*** | **Stroop**  **Priming Time (s)** |
| --- | --- | --- | --- | --- | --- | --- | --- |
| ALS | All | 38 | 25 | 13 | 60.7 ± 10.2 | 316 ± 458 | 65.1 ± 14.1 |
|  | Spinal | 31 | 23 | 8 | 61.3 ± 10.7 | 359 ± 494 | 63.3 ± 13.8 |
|  | Bulbar | 6 | 2 | 4 | 56.2 ± 6.7 | 73 ± 35 | 75.2 ± 13.6 |
|  | ALS-FTD | 1 | 0 | 1 | 68.5 ± 0.0 | 442 ± 0 | 61.0 ± 0.0 |
|  | *C9ORF72*+ | 2 | 1 | 1 | 64.1 ± 6.3 | 445 ± 4 | 59.0 ± 2.8 |
|  | *C9ORF72*- | 36 | 24 | 12 | 60.5 ± 10.4 | 309 ± 470 | 65.4 ± 14.4 |

* mean ± standard deviation
